# Scientific faith: Belief in science increases in the face of stress and existential anxiety^☆^

**DOI:** 10.1016/j.jesp.2013.05.008

**Published:** 2013-11

**Authors:** Miguel Farias, Anna-Kaisa Newheiser, Guy Kahane, Zoe de Toledo

**Affiliations:** a Department of Experimental Psychology, University of Oxford, South Parks Road, Oxford OX1 3UD, England, UK; b Department of Psychology, Yale University, 2 Hillhouse Avenue, New Haven, CT 06511, USA; c Oxford Uehiro Centre for Practical Ethics, University of Oxford, Suite 8, Littlegate House, St. Ebbe's Street, Oxford OX1 1PT, England, UK

**Keywords:** Belief, Belief in science, Religious belief, Stress, Existential anxiety

## Abstract

Growing evidence indicates that religious belief helps individuals to cope with stress and anxiety. But is this effect specific to supernatural beliefs, or is it a more general function of belief — including belief in science? We developed a measure of belief in science and conducted two experiments in which we manipulated stress and existential anxiety. In Experiment 1, we assessed rowers about to compete (high-stress condition) and rowers at a training session (low-stress condition). As predicted, rowers in the high-stress group reported greater belief in science. In Experiment 2, participants primed with mortality (vs. participants in a control condition) reported greater belief in science. In both experiments, belief in science was negatively correlated with religiosity. Thus, some secular individuals may use science as a form of “faith” that helps them to deal with stressful and anxiety-provoking situations.

## Highlights

• We predicted that stress and existential anxiety would result in greater belief in science.
• Athletes about to compete (vs. training) reported greater belief in science.
• Mortality salience increased belief in science but not in scientific determinism.
• Secular individuals benefit from believing in science.

## Introduction

Beliefs matter. Our beliefs can comfort us, provide us with meaning, or tie us more closely to others (Heine, Proulx, & Vohs, 2006; Hogg & Mulling, 1999; Lerner & Miller, 1978). Authors such as Durkheim and Freud famously argued that religious belief plays such a role in the lives of believers. Recent research has provided evidence for this view, showing that religious belief can compensate for lack of control (Kay, Whitson, Gaucher, & Galinsky, 2009), alleviate anxiety (Inzlicht, Tullett, & Good, 2011; Norenzayan & Hansen, 2006), and relieve stress (Ano & Vasconcelles, 2004). What remains unclear, however, is whether these compensatory effects of religious belief are driven by its supernatural or transcendent content, or whether these effects instead stem from belief more generally. Accordingly, the aim of the present research was to investigate the relationship of stress and existential anxiety with non-religious beliefs of an avowedly naturalistic character — *belief in science*.

Religious practice and affiliation are on the decline in many Western countries. For instance, in the latest UK census, “no religion” was the second largest response category to an item assessing religious affiliation (Office of National Statistics, 2012). Although non-religious individuals also face stressful and anxiety-provoking situations, it is unlikely that they respond to such situations by appealing to beliefs with explicitly religious or transcendent content in the way religious believers can (e.g., Arndt, Greenberg, & Cook, 2002). An important question is thus what beliefs, if any, play a parallel compensatory role in the lives of secular individuals.

Some have suggested that, in the absence of religious belief, secular beliefs such as Humanism and various political ideologies can replace religion as a source of comfort and meaning (Gray, 2004; Popper, 1945/2003; Sartre, 1946). In line with this notion, recent studies have suggested that belief in human progress can serve the same compensatory functions previously implicated in religious belief (Rutjens, van der Pligt, & van Harrevald, 2009; Rutjens, van Harrevald, & van der Pligt, 2010). More controversially, it is sometimes argued that science itself can be the object of attitudes resembling religious faith. For example, some scientists and philosophers are accused of “scientism” and are claimed to have a dogmatic faith in scientific methods and results (Stenmark, 2001). Setting aside the merit of these accusations, it is clear that individuals differ in their attitudes toward science. Whereas most individuals accept science as a reliable source of knowledge about the world, only some perceive science as a superior, even exclusive, guide to reality, and as possessing a unique and central value (Haught, 2005; Sorell, 1991). We refer to such attitudes as belief in science. Such an allegiance to science often involves the categorical rejection of anything supernatural, and is thus typically in tension with religious belief. At the same time, religion and science share motivational similarities: Scientific ideas can be a source of meaning (Preston, 2012) and generate feelings commonly associated with religion, such as awe (Rogers, 2004; Sagan & Druyan, 2006). It is thus of particular interest whether belief in science can serve, in the secular context, the same comforting role that has been associated with religious belief.

We hypothesized that belief in the value of science as an institution and in its superiority as a source of knowledge can offer reassurance to secular individuals in threatening contexts. We therefore expected that situations that increase stress and existential anxiety — two constructs associated with a range of physiological, affective, and cognitive responses to threat (Greenberg, Solomon, & Pyszczynski, 1997; Kudielka & Kirschbaum, 2001) — would increase belief in science. To test these predictions, we developed a scale measuring belief in science and conducted two experiments in which we manipulated levels of stress or existential anxiety. Given that belief in the primacy of science is in tension with supernatural explanations, we also anticipated that belief in science would be negatively associated with religiosity.

## Experiment 1

We developed a scale assessing belief in science and carried out a field study comparing a group of rowers about to compete (high-stress condition) with a group of rowers who were training (low-stress control condition). We predicted that rowers in the high-stress condition would report greater belief in science.

## Method

### Scale development

We developed a 10-item scale that described ideas about science (see Table 1). The items were rated (1 = *strongly disagree* to 6 = *strongly agree*) by a sample of 144 participants (92 women; mean age = 24, *SD* = 7.54, range: 18–64), none of whom completed the main study. Exploratory factor analysis with Varimax rotation yielded one factor that accounted for 57% of the variance. All ten items loaded on this factor (loadings ≥ .56; see Table 1). The scale had high internal reliability, α = .86. Overall, this sample reported moderate levels of belief in science, *M* = 3.23, *SD* = 1.04.

### Participants and procedure

One hundred participants (46 women; mean age = 23, *SD* = 4.18, range: 16–43) were recruited for the main study through rowing coaches and athletes. Fifty-two participants were tested 35–45 min before competing in a rowing regatta (Metropolitan Regatta, Marlow Regatta or Henley Women's Regatta), representing the high-stress group. The control (low-stress) group (*N* = 48) was tested at a training session. Across both groups, athletes were of an amateur international standing (i.e., routinely took part in international competitions and trained on average 6 days per week). The questionnaire included one item measuring stress (“How much stress do you feel at this moment?”; 1 = *no stress at all* to 7 = *very much*), the new 10-item Belief in Science Scale (α = .87), and one item assessing religiosity (“How religious do you consider yourself to be?”; 1 = *not at all* to 7 = *very much*).

## Results and discussion

As intended, rowers about to compete were experiencing more stress (*M* = 4.04, *SD* = 1.36) than rowers at a training session (*M* = 3.02, *SD* = 1.76), *t*(98) = 3.26, *p* = .002, *d* = 0.66. Attesting to the secular nature of the sample, participants reported a very low degree of religious commitment (*M* = 1.86, *SD* = 1.69); religiosity did not differ between conditions, *p* = .225. As expected, belief in science was negatively correlated with religiosity, *r*(98) = − .29, *p* = .004.

Of primary interest, and as predicted, rowers in the high-stress condition reported greater belief in science (*M* = 4.03, *SD* = 0.87) than rowers in the low-stress condition (*M* = 3.54, *SD* = 0.86), *t*(98) = 2.82, *p* = .006, *d* = 0.57. Thus, the novel measure of belief in science differentiated between individuals facing different levels of stress. The greater belief in science observed in the high-stress condition is consistent with the notion that belief in science may help secular individuals to cope with stress.

We acknowledge that alternative explanations for increased belief in science in the high-stress condition are also possible. For example, rowers about to compete (vs. rowers in training) may have been more motivated to consider their scientific-based training regimen or equipment. However, we also note that training regimens and equipment may, in fact, be more salient during training sessions (which usually revolve around such regimens and equipment).

## Experiment 2

Experiment 2 extended Experiment 1 in several ways. First, we manipulated existential anxiety (rather than stress) in a more controlled experimental setting, using the mortality salience paradigm. A large body of research has established that being reminded of one's own death (“mortality salience”) results in existential anxiety, which leads people to defend their belief systems (e.g., Greenberg et al., 1997). Although it has been argued that, in the context of ideas about death, science may not be as comforting as religion and that in such contexts non-believers may resort to religious concepts (Inzlicht et al., 2011), recent research revealed that mortality salience did not increase supernatural beliefs in an atheist sample (Vail, Arndt, & Abdollahi, 2012). Accordingly, we hypothesized that, within our secular sample, mortality salience would increase belief in science (but would not affect religiosity). By using a different experimental manipulation from Experiment 1, we sought to show that the compensatory role of belief in science could be generalized to other threatening contexts.

Second, we investigated whether the effect observed in Experiment 1 was specific to belief in science, or whether it also generalizes to more particular views associated with modern science. To address this issue, we explored a different set of science-related ideas, *scientific determinism*, which reflects the extent to which people believe that their behavior is shaped and determined by nature, genes, and the environment, as opposed to their own volition (Paulhus & Carey, 2010), and is measured with items such as “Your genes determine your future” and “Childhood environment will determine your success as an adult.” Although the popular imagination often associates such deterministic claims with modern science, scientific determinism and belief in science are distinct constructs. Scientific determinism focuses exclusively on the biological and environmental factors that shape how people act. It is a view, not about science, but about the world, and indeed one that some find disturbing rather than comforting (Pinker, 2008). By contrast, belief in science indexes the belief that scientific inquiry is a method and form of knowledge, superior to all others, that allows us to understand the world. It is therefore likely that individuals derive greater meaning and purpose from believing in the value of science, than in causal determinants, even if scientific-based, of human behavior. Accordingly, although we anticipated a positive association between belief in science and scientific determinism, we nevertheless hypothesized that mortality salience would increase belief in science, but not scientific determinism.

Finally, we added a measure of spirituality, given that some individuals may maintain supernatural beliefs even after rejecting organized religion. We predicted that both religiosity and spirituality would be negatively associated with belief in science.

## Method

### Participants

Sixty participants (24 women; mean age = 31, *SD* = 12.47, range: 17–81) were recruited among staff and students at two large UK universities and were reimbursed for participating (£5 and a prize drawing for a £100 Amazon voucher).

### Procedure

Participants were randomly assigned to the mortality salience condition, *N* = 31, or the control condition, *N* = 29. Following prior work on mortality salience (e.g., Greenberg et al., 1997), participants in the mortality salience condition began the study by writing about the thoughts and feelings aroused by thinking about their own death, whereas in the control condition participants wrote about experiencing dental pain. Participants next completed the Positive and Negative Affect Schedule (Watson, Clark, & Tellegen, 1988), assessing positive (10 items; α = .87) and negative affect (10 items; α = .90). Our aim was to provide a delay between the mortality salience manipulation and the dependent measures (see Greenberg, Pyszczynski, Solomon, Simon, & Breus, 1994) and to ensure that participants' mood did not differ between the experimental conditions.

Participants then completed (1 = *strongly disagree* to 6 = *strongly agree*) scales assessing belief in science (α = .88) and scientific determinism (Paulhus & Carey, 2010; seven items, α = .73).

Finally, three items measured religiosity (e.g., “How religious do you consider yourself to be?”; α = .84) and three items measured spirituality (e.g., “How spiritual do you consider yourself to be?”; α = .81). Participants were also asked to note down any thoughts on the purpose of the study (none guessed its true purpose).

## Results and discussion

There were no differences between the two conditions on positive and negative affect, *p*s = .110 and .965, respectively. There were also no differences between the two conditions on religiosity, *p* = .823, or spirituality, *p* = .769. Overall, participants scored low on both religiosity (*M* = 1.81, *SD* = 1.11) and spirituality (*M* = 1.98, *SD* = 1.29). As expected, belief in science was negatively correlated with both religiosity, *r*(58) = − .51, *p* < .001, and spirituality, *r*(58) = − .48, *p* < .001.

Belief in science and scientific determinism were positively correlated, *r*(58) = .42, *p* = .001. Given the moderate size of this correlation, we conducted a principal components analysis (Oblimin rotation) on items assessing belief in science and scientific determinism, which suggested the presence of three factors. The first factor (eigenvalue = 5.74) consisted of the ten belief in science items (loadings ≥ .62). The second factor (eigenvalue = 2.02) consisted of three scientific determinism items (referring to environmental factors as determining behavior; loadings ≥ .68). The final factor (eigenvalue = 1.79) consisted of the remaining four scientific determinism items (referring to biological factors as determining behavior; loadings ≥ .66). Although scientific determinism loaded on two factors, the full scale had acceptable internal consistency, α = .73, and we therefore averaged the seven items into one index of scientific determinism. Together, the correlation and the principal component analysis confirm our reasoning that belief in science and scientific determinism are related, yet distinct, constructs.

Our main analysis was a multivariate analysis of variance (MANOVA), with experimental condition as a between-subjects factor and belief in science and scientific determinism as the dependent measures. The multivariate effect of condition approached significance, *F*(2, 57) = 2.80, *p* = .069, η^2^*_p_* = .09. Supporting our primary prediction, univariate analyses revealed that participants reported significantly greater belief in science in the mortality salience condition (*M* = 3.94, *SD* = 1.04) than in the control condition (*M* = 3.41, *SD* = 0.67), *F*(1, 58) = 5.39, *p* = .024, η^2^*_p_* = .09. In contrast, as anticipated, the effect of condition on scientific determinism was nonsignificant, *F*(1, 58) = 1.99, *p* = .164, η^2^*_p_* = .03 (mortality salience condition: *M* = 3.44, *SD* = 0.74; control condition: *M* = 3.19, *SD* = 0.66).

## General discussion

Science and religion are often taken to offer competing explanations of the world (Preston & Epley, 2009). That science can be a source of meaning, similar to religion, is not a completely new idea; it has been raised by philosophers (Ziman, 1978/1991) and scientists (Dawkins, 1997) alike. While many have attempted to understand the emotional or social underpinnings of religious belief, the possibility that science might serve similar psychological functions has received less attention. Employing a novel field experiment and a well-researched experimental paradigm, our two experiments indicate that belief in science increases when individuals are placed in threatening situations. Our findings suggest that belief in science may help non-religious people deal with adverse conditions, as has been reported previously for religious belief (Inzlicht et al., 2011; Kay et al., 2009; Norenzayan & Hansen, 2006), belief in progress (Rutjens et al., 2009; Rutjens, van der Pligt, & van Harreveld, 2010), and belief in intelligent design and evolutionary theory (Rutjens, van der Pligt, et al., 2010; Tracy, Hart, & Martens, 2011). We acknowledge, however, that we examined only one direction of the effect; investigating whether affirming one's belief in science indeed reduces stress and existential anxiety thus represents a particularly productive direction for future research.

We acknowledge a further limitation, which may in fact inspire future research in fruitful ways: In Experiment 2, we did not observe an effect of mortality salience on religiosity, which is in apparent contrast with previous work that has found an increase in religiosity after a mortality prime (e.g., Norenzayan & Hansen, 2006). We suggest two reasons for this null effect: First, we assessed self-perceived religiosity and extent of religious practice, while other studies on mortality salience have used measures of religious *belief*; our measure was, thus, likely not sensitive to changes in religious belief. Second, and more importantly, our participants were largely secular (e.g., in Experiment 2, 51 out of 60 participants scored below the scale midpoint on religiosity). Mortality salience is expected to activate beliefs that are relevant to one's worldview (e.g., Rosenblatt, Greenberg, Solomon, Pyszczynski, & Lyon, 1989), and should thus not activate religiosity among secular individuals (Vail et al., 2012). Future research would benefit from testing secular and religious populations to directly compare the strength, and ascertain the mediating mechanisms, of the compensatory effects of scientific and religious belief.

The relationship between belief in science and belief in progress also needs to be addressed. Is belief in science a belief in a human institution that is continually advancing, which would make it a specific case of belief in progress; or is it belief in a method that allows us to make sense of the world? Contrary to belief in progress, which is laden with a sense of positive hope and is therefore essentially an evaluative concept (Rutjens et al., 2009; Rutjens, van der Pligt, et al., 2010), belief in science is largely an epistemic worldview, expressing confidence in a distinctive method for understanding the world. The two notions need not overlap: One can have confidence in science, yet hold a deeply pessimistic view of the future. Conversely, there is no epistemic component to belief in progress, which is associated with moral progress and is, in principle, compatible with belief in the supernatural.

It is perhaps not surprising that secular belief systems like Humanism and belief in progress can play a comforting role, as they present the world as a broadly moral order. By contrast, our findings suggest that merely believing in the superiority of science as a method of making sense of the universe may be sufficient to play such a compensatory role, even if the order that science reveals is not moral, and perhaps independently of any optimism about the future.

The suggested parallels between religious belief and belief in science may seem to be in tension with recent work emphasizing the intuitive character of religious belief. Tasks involving more analytic processing were shown to decrease religious belief (Gervais & Norenzayan, 2012), whereas the stimulation of a more intuitive mindset led to a greater belief in God (Shenhav, Rand, & Greene, 2012). Contrary to religion, scientific practice is defined by analytical thinking; rational enquiry and weighing of evidence are given precedence even when they conflict with intuition. But when it comes to believing, even if it is a belief in the scientific method as opposed to divine revelation, the underlying mechanism may be similar. Despite their different methods, both science and religion offer powerful explanations of the world (Preston & Epley, 2005), which may work at an intuitive level to provide comfort and assurance. That modern secular individuals are prone to cling on to beliefs about science, in the same way that their ancestors turned to the gods, carries no judgment on the value of science as a method but simply highlights the human motivation to believe.

## Figures and Tables

**Table 1:** Belief in science scale: Items and factor loadings from the scale validation sample.

Item	Loading
Science provides us with a better understanding of the universe than does religion.	.76
“In a demon-haunted world, science is a candle in the dark.” (Carl Sagan)	.73
We can only rationally believe in what is scientifically provable.	.73
Science tells us everything there is to know about what reality consists of.	.78
All the tasks human beings face are soluble by science.	.71
The scientific method is the only reliable path to knowledge.	.84
The only real kind of knowledge we can have is scientific knowledge.	.83
Science is the most valuable part of human culture.	.77
Science is the most efficient means of attaining truth.	.83
Scientists and science should be given more respect in modern society.	.56
